# A systematic review on the burden of illness in individuals with tuberous sclerosis complex (TSC)

**DOI:** 10.1186/s13023-019-1258-3

**Published:** 2020-01-21

**Authors:** Johann Philipp Zöllner, David Neal Franz, Christoph Hertzberg, Rima Nabbout, Felix Rosenow, Matthias Sauter, Susanne Schubert-Bast, Adelheid Wiemer-Kruel, Adam Strzelczyk

**Affiliations:** 1 Epilepsy Center Frankfurt Rhine-Main and Department of Neurology, Goethe-University Frankfurt, Frankfurt am Main, Germany; 2 LOEWE Center for Personalized Translational Epilepsy Research (CePTER), Goethe-University Frankfurt, Frankfurt am Main, Germany; 3 Children’s Hospital Medical Center, University of Cincinnati College of Medicine, Cincinnati, OH USA; 4 Zentrum für Sozialpädiatrie & Neuropädiatrie (DBZ), Vivantes Klinikum Neukölln, Berlin, Germany; 5 Department of Pediatric Neurology, Necker Enfants Malades Hospital, Paris Descartes University, Imagine Institute UMR1136, Paris, France; 6 Klinikverbund Kempten-Oberallgäu gGmbH, Kempten, Germany; 7 Department of Neuropediatrics, Goethe-University Frankfurt, Frankfurt am Main, Germany; 8 Clinic for Children and Adolescents, Epilepsy Center Kork, Kehl-Kork, Germany; 9 Epilepsy Center Hessen and Department of Neurology, Philipps-University Marburg, Marburg (Lahn), Germany; 10 Epilepsy Center Frankfurt Rhine-Main, Center of Neurology and Neurosurgery, Goethe-University Frankfurt, Schleusenweg 2-16 (Haus 95), 60528 Frankfurt am Main, Germany

## Abstract

**Objective:**

This review will summarize current knowledge on the burden of illness (BOI) in tuberous sclerosis complex (TSC), a multisystem genetic disorder manifesting with hamartomas throughout the body, including mainly the kidneys, brain, skin, eyes, heart, and lungs.

**Methods:**

We performed a systematic analysis of the available literature on BOI in TSC according to the PRISMA guidelines. All studies irrespective of participant age that reported on individual and societal measures of disease burden (e.g. health care resource use, costs, quality of life) were included.

**Results:**

We identified 33 studies reporting BOI in TSC patients. Most studies (21) reported health care resource use, while 14 studies reported quality of life and 10 studies mentioned costs associated with TSC. Only eight research papers reported caregiver BOI. Substantial BOI occurs from most manifestations of the disorder, particularly from pharmacoresistant epilepsy, neuropsychiatric, renal and skin manifestations. While less frequent, pulmonary complications also lead to a high individual BOI. The range for the mean annual direct costs varied widely between 424 and 98,008 International Dollar purchasing power parities (PPP-$). Brain surgery, end-stage renal disease with dialysis, and pulmonary complications all incur particularly high costs. There is a dearth of information regarding indirect costs in TSC. Mortality overall is increased compared to general population; and most TSC related deaths occur as a result of complications from seizures as well as renal complications. Long term studies report mortality between 4.8 and 8.3% for a follow-up of 8 to 17.4 years.

**Conclusions:**

TSC patients and their caregivers have a high burden of illness, and TSC patients incur high costs in health care systems. At the same time, the provision of inadequate treatment that does not adhere to published guidelines is common and centralized TSC care is received by no more than half of individuals who need it, especially adults. Further studies focusing on the cost effectiveness and BOI outcomes of coordinated TSC care as well as of new treatment options such as mTOR inhibitors are necessary.

## Introduction

Tuberous sclerosis complex (TSC) is a rare genetic disorder that affects about 1 in 5000 individuals worldwide [1–7]. Its prevalence was until recently underestimated due to incomplete penetrance and the considerable interindividual phenotypic variability in individuals with TSC [8, 9]. TSC can affect many organs, leading to benign tumors presenting preferentially in the skin, brain, and kidneys. The clinical manifestation of the disorder changes during life in a typical pattern. Many individuals are first diagnosed by pathognomonic skin manifestations or secondarily after experiencing seizures, as most individuals with TSC are affected by a structural epilepsy due to cortical tubers or other cortical malformations. The clinical picture of TSC is very broad and can range from mild symptoms that do not limit the individual to manifestations with severe disabilities in multiple organ systems, often involving intellectual impairment.

TSC is caused by mutations in the *TSC1* or *TSC2* gene. It is inherited in an autosomal-dominant fashion, but most cases are due to apparent de novo mutations. Genetic mosaicism and deep intronic mutations probably contributes to the disorder in the 15% of individuals where no definitive hereditary mutation can be found despite a definite clinical diagnosis of TSC [10].

Burden of illness (BOI) describes the impact of a health problem on the individual and society as a whole. BOI includes an epidemiological domain which encompasses both the years of life lost due to the disease (mortality) as well as the morbidity, which refers to disease prevalence and associated years with reduced health. The economic domain of the BOI comprises direct and indirect costs as well as health care resource utilization [11]. Direct costs reflect costs to the individual or health care system and can be easily quantified (e.g. co-payments, cost of hospital admission) [12]. In contrast, indirect costs contain financial and social burden to the individual and his surroundings (e.g. a parent’s time lost from work) and may be less quantifiable [13, 14]. Health care utilization reflects resources used by the patient or his caregivers in an inpatient or outpatient setting, including medication and other medical treatment (e.g. physiotherapy, logopedic therapy). Individual BOI is usually expressed as quality of life (QoL) and is measured by standardized questionnaires.

The burden of illness in TSC is highly variable and determined by the condition’s complex and multifaceted disorder manifestations. These manifestations and their clinical significance vary widely between persons with TSC as well as throughout individual’s lifetime. In addition, the relevance of specific manifestations may be assessed differently between the individual with TSC, his/her caregivers, and the treating health care providers – a general problem when evaluating the BOI in any disease with medical or economic approaches.

The first comprehensive review on the burden of illness in TSC was published by Hallett et al. in 2011 [15], with the majority of studies on this topic published in the time since then. An outstanding review on genetic, clinical, and therapeutic aspects of TSC was presented by Henske et al. in 2016 [16], but BOI was not addressed in this review in detail.

Thus, the present article aims to give a systematic review of the known factors that contribute to the BOI in individuals with TSC and their caregivers. The clinical picture of TSC is summarized in order to aid interpretation of the health burden.

## Materials and methods

We performed a structured analysis of the literature according to the Preferred Reporting Items for Systematic Reviews and Meta-analyses (PRISMA) statement [17]. We included 33 articles covering both children and adults with TSC which reported data on BOI in TSC in the systematic review, while information from these and further articles was used for a non-systematic clinical summary. Studies included featured noninterventional retrospective, prospective, and cross-sectional as well as interventional designs. We defined the following relevant parameters: incidence and prevalence of organ system manifestations; individual morbidity and mortality (described as disease-adjusted life years (DALY) and quality-adjusted life years (QALY), where available); resource utilization of health care systems (described in total use of health care resources) and direct as well as indirect costs. Caregiver burden was assessed as well. Costs were extracted as given in the source and then converted into 2018 International Dollar purchasing power parities (PPP-$) according to the method described by Strzelczyk et al. [18]. In short, inflation data were retrieved for each country from the Organization for Economic Co-Operation and Development (OECD) Stat database [19]. PPP were defined as the rates of currency conversion that eliminate the differences in price levels between countries. PPP conversion factors were obtained from the OECD Stat database [19].

### Search strategy

The online databases PubMed and MEDLINE as well as the Cochrane Library were searched using the search string *“TSC OR tuberous sclerosis complex AND (burden of illness OR BOI OR health care use OR health care utilization OR health care utilisation OR resource use OR resource utilization OR resource utilisation OR economic burden OR health burden OR health care costs OR costs OR disease-adjusted life years OR DALY OR quality-adjusted life years OR QALY OR quality of life)”*. Additionally, we searched using the following PubMed medical subject heading (MeSH) terms: *(“Tuberous Sclerosis/economics”[Mesh] OR “Tuberous Sclerosis/epidemiology”[Mesh] OR “Tuberous Sclerosis/statistics and numerical data”[Mesh])*. In addition, the references of included studies were scanned to identify further suitable articles. We restricted the analysis to articles published in indexed, peer-reviewed, journals until October 2019 and which were available through usual library services such as digital and printed records and repositories. Only studies written in English were included in the final evaluation. The last search was performed on October 17, 2019.

**Fig. 1:**
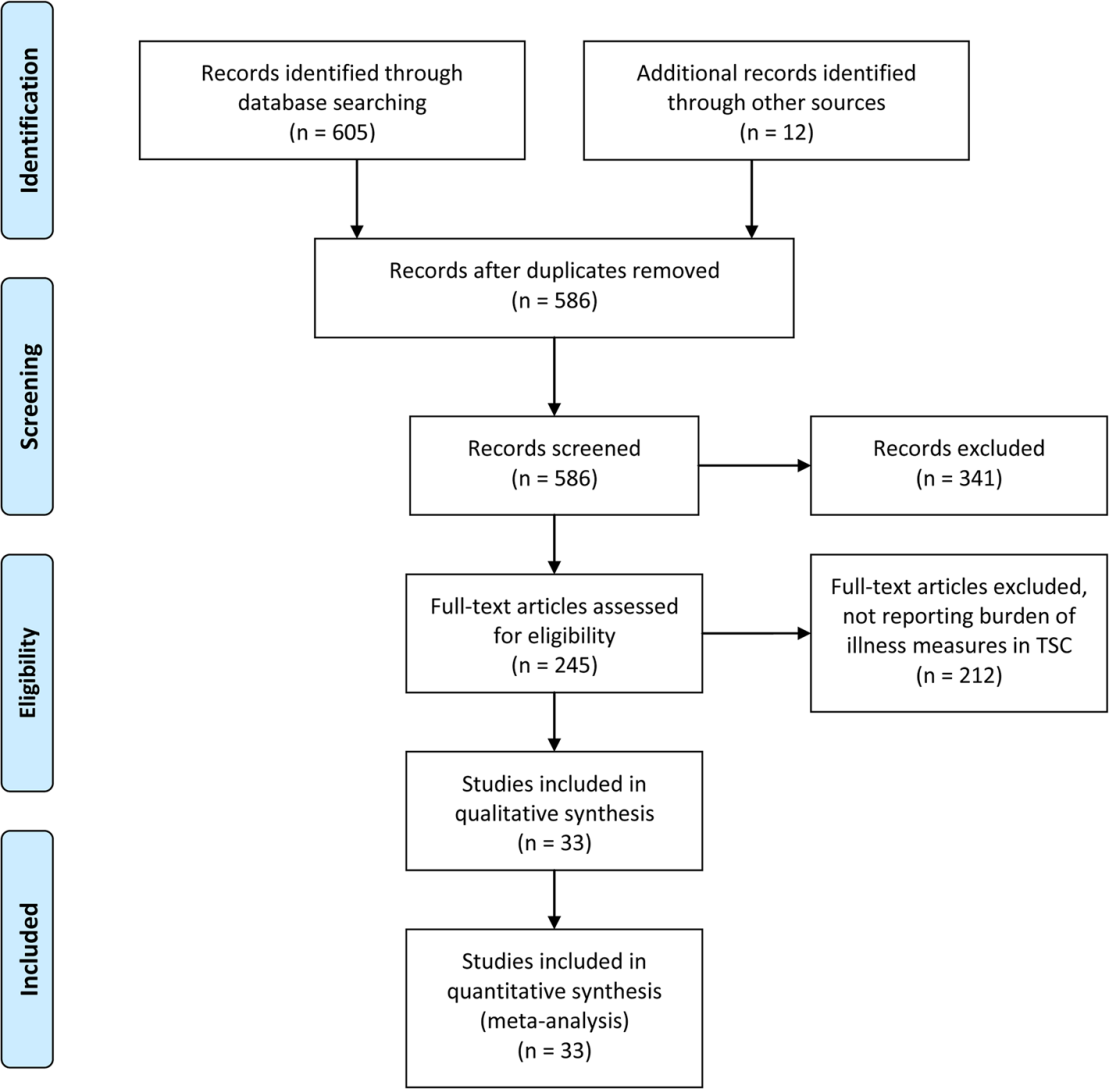
Results from the systematic literature search according to the Preferred Reporting Items for Systematic Reviews and Meta-Analyses (PRISMA) guidelines

All studies were screened for eligibility. The initial search returned a total of 605 papers (359 by using PubMed keywords, 245 by using MEDLINE MeSH terms, and one by using the Cochrane keyword search). An additional 12 papers were found by searching the literature references, for a total of 617 papers. Following the removal of 31 duplicates (including the single paper found through the Cochrane search), 586 papers remained. The titles and abstracts of the remaining studies were screened and 341 studies were removed, as they were deemed to be not within the general scope of this review. The remaining 245 studies were evaluated based on the details of their respective full texts. Of those, 212 papers were eventually removed based on not reporting at least one BOI measure (see Fig. 1 for details). In total, 33 studies were included in the systematic part of this review.

Of note, merging absolute patient numbers from different studies is challenging because of a possible overlap of individuals, especially in studies recruiting individuals from similar sources, such as patient support groups or studies from the same groups or countries.

## Results

### Organ manifestations

**Fig. 2:**
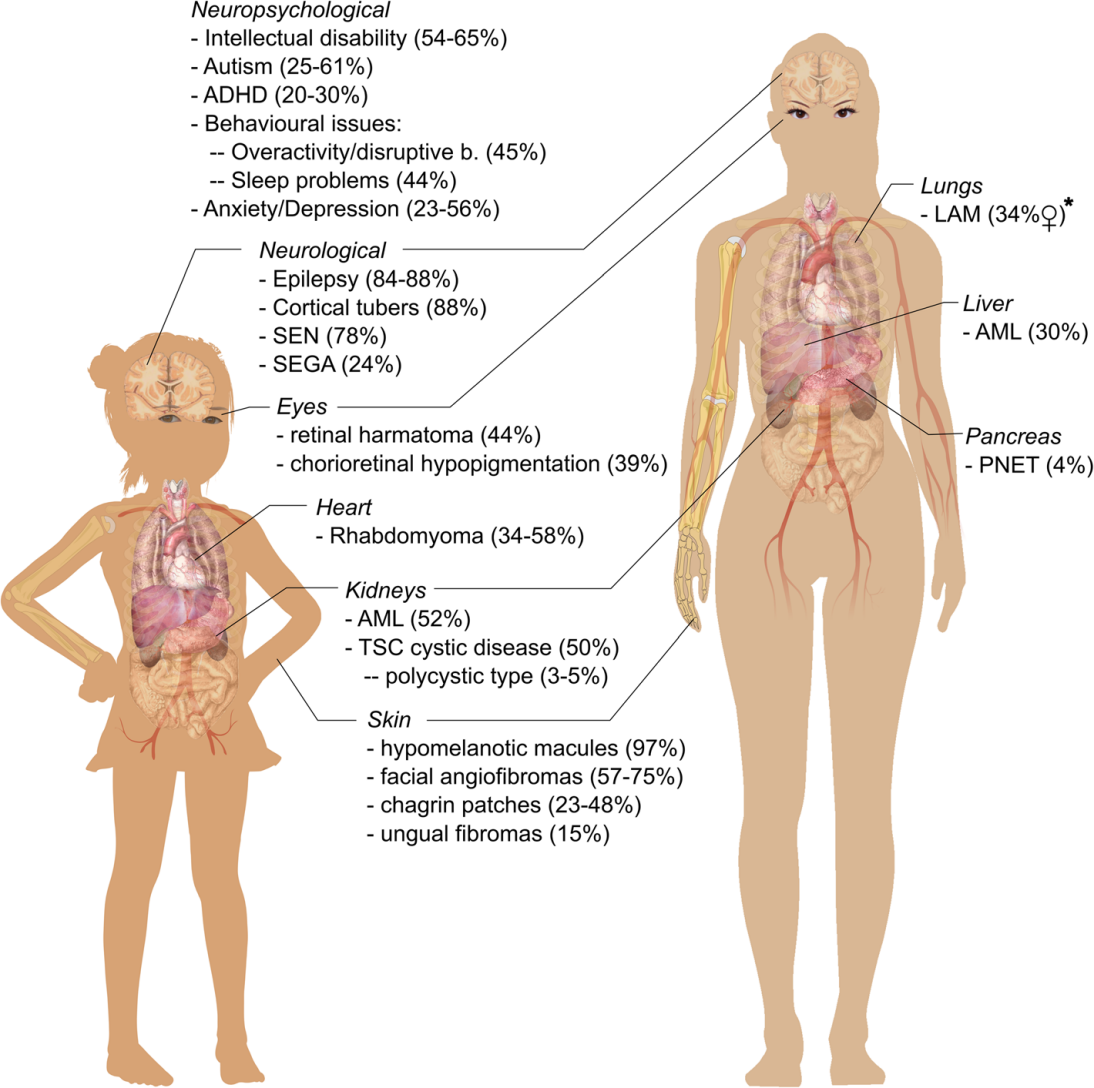
Graphical overview of clinical organ manifestations in Tuberous Sclerosis Complex (TSC). Prevalences are given in parentheses after each manifestation. *Prevalence of LAM is given for female patients as there is a high gender-dependency. Prevalences for the majority of manifestations change with age. For sources of the prevalences, please refer to section 3.1 of the manuscript. ADHD = attention deficit hyperactivity disorder, AML = angiomyolipoma, LAM = lymphangioleiomyomatosis, PNET = pancreatic neuroendocrine tumor, SEGA = subependymal giant astrocytoma, SEN = subependymal nodules. Graphic adapted from “Female_shadow_template.svg” and “Girl_diagram_template.svg”, available in the public domain and accessible at https://commons.wikimedia.org/wiki/Human_body_diagrams (original author: Mikael Häggström)

Individuals with TSC experience a variety of organ manifestations. The following paragraphs give a non-systematic clinical overview of TSC manifestations as a basis for further description of the specific BOI in TSC. For specifics on the manifestations, please refer to Table 1. For a graphic overview, please refer to Fig. 2. Wherever possible, we refer to systematic reviews for certain manifestations.

**Table 1:** Prevalence of common clinical manifestations in TSC patients

Clinical category	Prevalence	Clinical Notes
Neurological manifestations		
Epilepsy	83.5–88.4% [20–25]	The prevalence rate (32.9%, 35.9%) of pharmacoresistant epilepsy is similar to general population with focal epilepsy [26, 27]. A mutation in the *TSC2* gene is a risk factor for infantile spasms (47.3% vs. 23% with TSC1 in TOSCA) as well as an earlier manifestation of epilepsy, a higher seizure frequency, and pharmacological refractoriness [28].
Epileptic spasms	38–49% [21, 22, 26]	Most children with TSC and West syndrome develop symptomatic generalized epilepsy (62%) [29, 30].
Cortical tubers	88.2% [20, 18, 24]	
Subependymal nodules (SEN)	78.2% [20, 18, 24]	
Subependymal giant cell astrocytoma (SEGA)	24.4% [20, 18, 31, 32]	SEGAs arise from serially growing SEN, are often greater than 1 cm in diameter, and are generally located near the foramina of Monro [33]. While SEGAs generally have a low incidence after adolescence, SEGA growth affected 21–29% past the second decade of life [20, 34] in two studies.
Neuropsychiatric manifestations (TSC-associated neuropsychiatric disorders [TAND])		
Intellectual disability	53.6–65% [1, 23, 35–37]	Joinson et al. [38] described a bimodal distribution of intellectual impairment in TSC. About two-thirds of the studied individuals had an intelligence quotient (IQ) in the normal range, albeit with an overall negative shift (mean IQ: 93), while 31% had a profound intellectual disability [38]. Individuals with severe intellectual disability due to TSC have higher levels of verbal disability that do those with severe intellectual disability from other causes [39]. Many individuals with TSC have more than one neuropsychiatric disorder [40, 41].
Autism	25–61% [21, 23, 24, 40, 42–47]	Intellectual impairment and the presence of infantile spasms are associated with higher risks for both autism and ADHD [36, 48].
Attention-deficit hyperactivity disorder (ADHD)	19.6–30% [21, 42, 45, 49]	
Behavioral problems	Overactivity 45% [50] Impulsivity 42.7% [50] Severe aggression 24.3% [50] Sleep issues 43.9% [50]	Rates of self-injury and aggression in adults with TSC with intellectual disability: 31 and 37.9%, respectively [51]. In TOSCA, significantly higher rates of overactivity and impulsivity were seen in children; in adults, higher rates of anxiety, depressed mood, mood swings, obsessions, psychosis and hallucinations were reported [50] The relationship between cortical tubers and autism spectrum disorders is mediated by general cognitive impairment [52].
Depression	23.4–56% [42, 45, 49, 53–55]	A UK study [42] showed that the depression rate among patients with TSC was not higher than that in a matched general population comparator cohort. A United States (US) study reported that individuals with TSC had significantly higher depressive symptom scores as compared with the general population (11.6 vs. 5.1 on the Hamilton Depression Inventory—short form) [56]. Depending on the scoring system used, 19% (Hospital Anxiety and Depression Scale; HADS) to 43% (Symptom Checklist-90-Revised) of adults with TSC present elevated depression scores [54, 55]. A study identified HADS scores suggesting anxiety in 56% of adult individuals with TSC [54]. A study on individuals with TSC in transition from pediatric treatment found frequent sadness and depression in 60% of patients and high anxiety in 40% [53]. Chung et al. [40] demonstrated in a retrospective analysis that behavioral problems and mood disorders can be successfully treated medically in about two-thirds of afflicted individuals.
Renal manifestations		
Angiomyolipoma (AML)	51.8% [21]	Recent publications from the TOSCA registry [57] have hinted at an even higher rate of AML than previously known (51.8% of 2216 individuals) and suggest an earlier onset in early childhood. A retrospective, longitudinal Dutch cohort study in 369 individuals with TSC and chronic kidney disease (CKD) or angiomyolipoma of the kidneys reported that during follow-up, 16% of patients achieved CKD stage 3 or higher [35]. A strong association between age, AML size, and CKD was observed. In a UK study [42], CKD (stages 3–5) was found more frequently in individuals with TSC of all ages than in the general population at the same age intervals. Of note, a peak in the patients over 65 years cohort (42%) was noted.
Renal cell carcinoma	1–2% [21, 58, 59]	Incidence is similar compared to the general population. Renal cell carcinoma can manifest earlier than in the general population, even in children and young adults.
TSC renal cystic kidney disease	Total 50% [60] Severe (Polycystic kidney disease, PKD) 3.5% [21]	PKD is a rare manifestation in TSC. The PKD1 gene is situated next to the *TSC2* gene on chromosome 16, so in rare cases a contiguous gene syndrome with severe polycystic kidney disease and early loss of renal function can develop in individuals with TSC. Milder, typically asymptomatic forms of TSC renal cystic disease without a certain link to PKD mutations are more common, more commonly in individuals with *TSC2* mutations [61].
Pulmonary manifestations		
Lymphangioleiomyomatosis (LAM)	34–81% of female individuals [62], rare in males	A Dutch study [62] identified LAM-typical cysts in 52 (28%) of 186 individuals with TSC. Pulmonary cysts were detected much more frequently in females (42%), but also in 13% of males [62]. In general, however, cysts were larger and more numerous in women than in men. Also, considerable cystic changes were detected almost exclusively in women (in 33 women versus in three men). Another study found LAM prevalence increasing rates in women with age (27% at the age of 21 years and 81% at the age of 40 years and older) [63]. A long-term LAM register study from the US showed 26 deaths and 43 lung transplantations occurred over a follow-up of 13 to 17 years in 217 patients. Diagnosis after menopause and better baseline lung function decreased transplantation probability or risk of death. Of note, only 36 of 217 patients had TSC-LAM. The presence of TSC-LAM did not significantly affect time to transplantation or death.
Cardiac manifestations		
Cardiac rhabdomyoma	34–58% [21]	Rhabdomyoma in TSC are typically, but not exclusively, multifocal.
Aortic aneurysm		Rare, but can develop from early age [64].
Cutaneous manifestations		
Hypopigmented macules (“Ash-leaf spots”)	66.7–97.2% [21, 65]	Detection can be eased by Wood light in persons with a light skin tone. Hypopigmented macules more rarely manifest as “Confetti-like” lesions (2.8% [65]).
Angiofibromas	57.3–74.5% [21, 65]	Usually appear from the 2nd to 5th year of life.
Chagrin patches	22.7–48.1% [21, 65]	Connective tissue hamartoma, mostly on dorsal body surfaces such as the lower back region.
Molluscum fibrosum pendulans	22.6% [65]	
Forehead plaque	18.9% [65]	
Periungual fibromas	15.1% [65]	Usually appear first in childhood/adolescence.
Ocular manifestations		
Retinal hamartomas	30–44% [24, 66]	
Chorioretinal hypopigmentation	39% [66]	
Other organ manifestations		
Hepatic (hepatic AML, hepatic cysts)	9.1% [21]	Associated with renal AML [67, 68]. These were found in 9.1% of individuals in TOSCA.
Pancreatic neuroendocrine tumors	4.1% [69]

#### Neurological manifestations

Epileptic seizures are a hallmark of TSC and the most common clinical manifestation. The majority of individuals (83.6%) in the multinational Tuberous Sclerosis Registry to Increase Disease Awareness (TOSCA) [20] had a diagnosis of epilepsy [21]. Prevalence can depend upon patients’ age and the extent of cerebral lesions. In general, seizures in patients with TSC are often detected before the third year of life^,^ [21, 22, 42], but can also develop for the first time in adulthood (up to 12% of cases). The most common seizure type in TSC are localization-related or focal seizures (67.5%), followed by epileptic (“infantile”) spasms in 38 to 49% [21, 22, 26] of individuals with TSC: Epileptic spasms typically begin between 4 and 8 months of age and later transform into other seizure types; they define West syndrome, a severe epileptic encephalopathy which is common in children with TSC. The majority of individuals with TSC characterized in the TOSCA registry had cortical tubers (88.2%) or subependymal nodules (SEN; 78.2%). Subependymal giant cell astrocytomas (SEGAs) were found in 24.4% of the TOSCA population. SEGA can obstruct the intraventricular space and lead to noncommunicating hydrocephalus requiring surgery (22.4% of those with SEGA in TOSCA) or shunt placement 4.1%) [21]. For a review on neurological manifestations of TSC, please see Lu et al. [70].

#### Neuropsychiatric manifestations

Cognitive, psychiatric, and neurodevelopmental problems are common in TSC, with one of these affecting almost all individuals with TSC at some point in their life. These different manifestations are grouped under the term “TSC-associated neuropsychiatric disorders” (TAND). Data from the TOSCA registry [21] revealed that 54.9% of persons with TSC had some level of intellectual disability (50 to 65% in prior studies [1, 23, 35–37]), with good agreement present between caregiver-reported and psychometric results. Generally, severe forms of intellectual disability are overrepresented in TSC [71]. Besides epileptic spasms, a prior history of seizures, greater seizure frequency, earlier seizure onset, and pharmacoresistant epilepsy all correlated with greater likelihood of developmental disabilities [22, 72–75]. Conversely, early seizure control improves the odds of a better cognitive outcome [76, 77]. TSC is one of the disorders most strongly connected with autism [78]. Rates of autism in individuals with TSC range up to 61% in smaller studies [43, 44]. Attention deficit hyperactivity disorder (ADHD) affects about 20–30% of individuals with TSC. On a behavioral level, the most common problems are, repetitive or disruptive behavior (13–56%) [45] and sleep issues (up to 60%) [46, 79]. Children with an intellectual disability are more likely to also exhibit behavioral problems, autism, ADHD, and speech difficulties [36]. Of note, a relevant proportion (about 30%) of intellectually unaffected children with TSC also exhibit behavioral disorders [36] or specific cognitive defects [80]. In a UK study [23], depression was diagnosed in about a quarter (23.4%) of adult patients. Individuals with TSC may also be more susceptible to stress-related disorders such as posttraumatic stress disorder [81]. The presence of a high proportion of missing data in the TOSCA-registered individuals suggests that TAND are in fact insufficiently diagnosed [21]. The use of a standardized TAND checklist is encouraged to improve the assessment of neuropsychiatric symptoms in TSC on, at minimum, a yearly basis [82]. For a succinct overview of TAND, please see Curatolo et al. [71], and for a recent review based on the TOSCA registry, please see de Vries et al. [50].

#### Renal manifestations

Renal angiomyolipomas (AML) occur in about one-half to two-thirds of individuals with TSC [61, 83–85]. They are believed to typically appear first in childhood and then tend to grow during adolescence and into adulthood [42]. The main complication of AML is retroperitoneal hemorrhage, which can be fatal due to the associated blood loss. The risk is greater as the tumors become larger. AML with a greatest diameter of more than 3 cm are frequently associated with dysplastic, aneurysmal blood vessels and carry an especially large bleeding risk. Other complications of AML may include pain, renal insufficiency, and arterial hypertension [83, 86]. The lifetime risk for renal hemorrhage ranges from 20% in women to 10% in men [87]. TSC renal cystic disease is seen in about half of individuals with TSC by MRI. Premature reduction of renal function affects almost half of individuals with TSC and is due to TSC renal cystic disease in addition to AML complications [60]. The risk of end-stage renal failure is presumed to be 1% in TSC [88, 89] and chronic kidney disease is an independent risk factor of cardiovascular diseases. Renal cell carcinoma (RCC) develops in about 2–4% of persons with TSC with a rate comparable to the general population [57–59]. RCC can however manifest earlier than in the general population. For a comprehensive review of TSC renal manifestations, please refer to Bissler and Kingswood [60].

#### Pulmonary manifestations

Lymphangioleiomyomatosis (LAM) is the primary pulmonic manifestation of TSC and almost exclusively affects adult females. The first diagnosis of LAM usually occurs around the end of the third decade of life, but insufficient diagnosis is a common problem in TSC-associated and spontaneous LAM [21, 42]. TSC-associated LAM can have very different consequences for the individual, ranging from radiological findings without clinical relevance to recurrent pneumothoraces with increased morbidity (hospital stays, pleurodesis or other operations) up to progressive loss of lung function. A US study found a prevalence of 34% among 38 women with TSC (mean age: 38 years; age range not given) who had no prior history of LAM.

#### Cardiac manifestations

Cardiac rhabdomyoma (CR) is one of the earliest clinical signs of TSC and can lead to a prenatal diagnosis of TSC on ultrasound [90]. In TOSCA, 34.3% of individuals had had CR. In a small study involving only 11 children [91], about one-third of the CR cases spontaneously regressed, and the CRs were rarely symptomatic (5.6% with arrhythmia/dysrhythmia and 2.9% with valve dysfunction). In another small study [92], TSC was diagnosed almost in all individuals who had a postnatal diagnosis of CR (*n* = 25), while either partial or complete regression was found in almost all children (96%). The authors suggested a likelihood of 79% for a TSC diagnosis later in life for individuals receiving a prenatal diagnosis of CR. Another small study [93] (*n* = 18) estimated this likelihood at 39%.

#### Cutaneous manifestations

Skin manifestations in TSC appear in nearly all individuals with TSC and can take many forms [24, 94]. Hypomelanotic macules (also seen in the form of larger “ash-leaf spots” or widespread “confetti macules”) are usually the first skin manifestation of TSC and appear in the majority of individuals with the disorder (prevalence of 66.7% in TOSCA), often at birth. Facial angiofibromas (AF) (prevalence of 57.3% in TOSCA) generally manifest in the first years of life (mean age of 6 years in TOSCA) and thereafter tend to grow. Less common are chagrin patches (prevalence of 22.7% in TOSCA), forehead plaques, and subungual fibromas. A US study [56] reported TSC-typical skin manifestations in 77% of children and 44% of adults. Enamel deficits are also common in TSC and can aid with clinical diagnosis [95].

#### Ocular manifestations

Retinal hamartomas are the most typical ocular manifestation of TSC. These are mostly asymptomatic, but can rarely impair vision. Generally, 30 to 40% of individuals with TSC are assumed to be impacted by hamartomas [24, 56, 66], with *TSC2* mutations conferring a particularly higher risk [96]. Further manifestations are achromatic patches (see Table 1).

#### Other organ manifestations

Usually asymptomatic hepatic manifestations (i.e., AML, cysts) are known in TSC [67] and are associated with renal AML [68]. These were found in 9.1% of individuals in TOSCA. Pancreatic neuroendocrine tumors, while overall rare (9/219; 4.1% in one study [69]), remain the most common pancreatic neoplasia in individuals with TSC [69]. Many more manifestations have been reported, such as sclerotic bone lesions [97] and cerebellar lesions in individuals with a TSC2 mutation [98] (see Table 1). The full scope of manifestations is however out of the scope of this non-systematic clinical overview.

In general, clinical hamartoma manifestations share a typical set of dynamics throughout an individual’s life. CRs, hypomelanotic macules, and seizures (and, in succession, SEN and cortical tubers) are often diagnosed in the first months or first years after birth. SEGA prevalence is highest during childhood. AML as well as some skin manifestations such as facial AF or chagrin patches appear in childhood, and data from TOSCA shows that their prevalence continues to increase into adulthood, while LAM and ungual fibromas are rarely diagnosed before adolescence. Cases of LAM are typically diagnosed at around 30 to 40 years of age in females.

### Burden of illness and resource use

Determining the burden of illness (BOI) of a disease requires summarizing its impact on the individual and society, while evaluating the use of health care resources (HCRU), morbidity, mortality, direct and indirect costs and individual and caregiver quality of life [18]. BOI can be expressed in health summary scores such as “disease-adjusted life years” (DALY), that represent years lost to disease from healthy life or “quality-adjusted life years” (QALY), which represents a compound of gained life expectancy and life quality in the setting of an interventional study (e.g. new drug). These measures do not reflect BOI on an individual level. On the ecnomic level, BOI consists of (1) direct costs to the health care system such as inpatient treatments or medications and (2) indirect costs due to a loss of productivity such as days off work, reduced working hours, or a job loss for the individual or caregivers. On an individual level, factors such as pain or discomfort or psychological stress of caregivers are included in quality of life measures. TSC’s wide phenotypic variety and changing clinical course throughout life leads to heterogeneous study cohorts with heterogeneous clinical manifestations. Thus, a complete evaluation of the BOI in TSC requires the assessment of a multitude of organ manifestations, making studies on BOI in TSC challenging to perform. Differences in health care accessibility and general organization of the health care system also hinder the aggregation of study results. Recently, de Vries et al. demonstrated that three health-related quality of life measures—the “Quality of Life in Childhood Epilepsy” (QOLCE), the “Quality of Life in Epilepsy Inventory for Adolescents-48” (QOLIE-AD-48), and the “Quality of Life in Epilepsy Inventory-31-Problems” (QOLIE-31-P) surveys—are largely applicable to individuals with TSC [99].

A total of 33 papers presented any data on BOI in TSC (Table 2 and Fig. 1). Of these, 21 papers report health care resource use [23, 24, 27, 35, 53, 56, 86, 104–108, 110–112, 114, 116, 118, 119, 120, 125]. Direct costs are reported by 10 studies [23, 35, 104, 106, 107, 110–112, 114, 119] and four studies assessed drivers of direct cost [23, 35, 110, 111]. The measures of DALY and QALY were only used by one study that calculated projected costs of different treatment strategies for epilepsy in TSC [109]. Fourteen studies reported on quality of life [53, 56, 101, 103, 105, 106, 108, 115, 118, 119, 121–123, 125]. Nine studies (also) reported BOI for caregivers [56, 102, 105, 106, 108, 115, 117, 119, 125] (five studies reported analyses of the same population or subgroups of the same). A total of 16 different questionnaires were used in individuals with TSC, none in more than four studies. In caregivers, six different questionnaires were used, not more than three in one study. The QOLCE and QOLIE were used in one study each [101, 103]. Please refer to Table 2 for details.

**Table 2:** Overview of studies reporting burden of illness by health care resource use, direct costs or mortality in TSC patients

Study	Type of study and data approach	Data source/ country of patient origin	Cohorts	Organ systems	Incl. / excl. Criteria	N total	N caregiver	Evaluated period (year)	Duration time (years)	Actual time evaluated (years)	Mean age	Mean age adults	Mean age children	N female (%)	N children (%)	Percentage caregiver	HRCU - inpatient	HRCU - outpatient	Direct costs	Cost drivers (regression analysis)	Social costs	Mortality	Individual BOI	Caregiver BOI
Shepherd et al. 1991 [100]	Retrospective chart review, bottom-up	Mayo Clinic/ USA	all TSC	all	Clinical diagnosis of TSC according to 1988 Gomez criteria	355	0	n. r.	n. r.	n. r.	n. r.	n. r.	n. r.	n. r.	n. r.	n. r.	n. r.	n. r.	n. r.	n. r.	n. r.	yes	only via mortality	n. r.
Liang et al. 2019 [101]	Prospective, single-center	Capital Epilepsy Therapy Center Beijing/ China	TSC + E	all	Pharmacoresistant epilepsy, surgery in year 2007	25	N/A	2007	1	1	14.3 (R 6–23)	n. r.	n. r.	8 (32)	100	N/A	n. r.	n. r.	n. r.	n. r.	n. r.	n. r.	Quality of life in epilepsy inventory-31 (QOLIE-31)	n. r.
Lennert et al. 2012 [24]	Retrospective chart review, bottom-up	Minnesota Epilepsy Group/ USA	only TSC + E	all	Inclusion: TSC–related seizure disorder within the first 6 months after tuberous sclerosis complex diagnosis exclusion: TSC–unrelated seizure disorder	95 (only children)	0	1973–2010	37	0.5 to 5	3.1 (SD 7.8)	N/A	3.1 (SD 7.8)	48 (51.0)	91 (95.8)	N/A	yes	yes	n. r.	n. r.	n. r.	n. r.	only via clinical information and HCRU	n. r.
Graffigna et al. 2013 [102]	Qualitative interview	survey of patients from 15 TSC centers/ Italy	Caregivers of children with TSC + SEGA	all	Caregivers of children with TSC + SEGA	0	48	2011–2012	1	N/A	47 (SD 6.3) (caregiver)	N/A	N/A	62.5 (caregiver)	N/A	100	n. r.	n. r.	n. r.	n. r.	n. r.	n. r.	n. r.	Qualitative statements
Krueger et al. 2013 [103]	Prospective, multicenter,open-label, phase!/ II clinical trial	TSC clinics at Cincinnati Children’s Hospital Medical Center (CCHMC) and Texas Children’s Hospital/ USA	Pharmacoresistant epilepsy	brain	> = 2 years of age, confirmed diagnosis of TSC (1998 clinical diagnostic criteria or identified disease-causing mutation, pharmacoreistant epilepsy)	23	N/A		16 months	16 months	8 (R 2–21)	n. r.	n. r.	50	n. r.	N/A	n. r.	n. r.	n. r.	n. r.	n. r.	n. r.	Quality of Life for Children with Epilepsy (QOLCE), Nisonger Child Behavioral Rating Form (NCBRF)	n. r.
Eijkemans et al. 2015 [86]	Retrospective observational, bottom-up	University Medical Center Utrecht (UMCU)/ Netherlands	all TSC/ TSC + AML	kidney	Diagnosis of TSC according to the modified Gomez criteria and aged 18 years or older	351^*^ (only adults)	0	1990–2012	22	median 15.8	39.8 (R 18–89)	39.8 (R 18–89)	N/A	175 (49.8)	N/A (0)	N/A	yes	n. r.	n. r.	n. r.	n. r.	yes	only via HCRU	n. r.
Kristof et al. 2015 [104]	Retrospective cohort study, top-down	Régie de l’Assurance-Maladie du Québec (RAMQ; Quebec Health Care Insurance Board) database/ Canada	all TSC/ TSC + LAM/ LAM/ controls	lung	LAM and/or TSC and controls	1004 (children and adults)	0	1996–2011	15	n. r.	39.5 (SD 24.4)	n. r.	n. r.	555 (55.3)	n. r.	N/A	yes	yes	yes	n. r.	n. r.	yes	only via HCRU	n. r.
Rentz et al. 2015a [56]	Cross-sectional, non-interventional, bottom-up	web-based survey/ USA	paediatric/ adult TSC patients/ caregivers	all	Inclusion: age 18 years or older, TSC diagnosis (or care for individual with TSC), read English and ability to participate, and consent exclusion: patients with cognitive impairment or other health impairment that interfere w/ survey completion	676^**^ (children and adults)	275 (179 adult caregiver, 96 pediatric caregiver)	2012	N/A	2 months (May and June 2012)	29.8 (SD 17.7)	38 (SD 12.8)	7.1 (SD 4.6)	400 (59.2)	197 (26.5)	40.7	yes	yes	n. r.	n. r.	n. r.	n. r.	Short Form (12) Health Survey (SF-12), Hamilton Depression Rating Scale-Short Form (HDI-SF)	SF-12,HDI-SF
Rentz et al. 2015b [105]	Cross-sectional, non-interventional, bottom-up	web-based survey/ USA	TSC caregivers	all	See Rentz et al. 2015a	275^**^ (children and adults)	275^**^	2012	0	2 months (May and June 2012)	n. r.	n. r.	n. r.	217 (79)	0	100	yes	yes	n. r.	n. r.	n. r.	n. r.	SF-12, HDI-SF	SF-12, HDI-SF
Skalicky et al. 2015 [106]	Cross-sectional, non-interventional, bottom-up	web-based survey/ USA	paediatric/ adult TSC patients	all	See Rentz et al. 2015 a	116^**^ (children and adults)	69^**^	2012	0	2 months (May and June 2012)	25.5 (SD 16.6)	36.8 (SD 11.5)	9 (SD 5.0)	n. r.	47 (40.5)	59.5	yes	yes	yes	n. r.	n. r.	n. r.	SF-12, HDI-SF, Work Productivity and Activity Impairment plus Classroom Impairment Questions: Special Health Problem (WPAI CIQ-SHP), version 2	SF-12, HDI-SF, WPAI CIQ-SHP-version 2
Sun et al. 2015 [107]	Retrospective cohort study, top-down	National claims databases (MarketScan commercial and Medicaid, Pharmetrics commercial)/ USA	all TSC + SEGA surgery	brain (SEGA)	TSC patients with first SEGA surgery claim in study period	47 (children and adults)	0	2000–2011	11	n. r.	11.6	n. r.	n. r.	16 (34)	n. r.	N/A	yes	yes	yes	yes	n. r.	n. r.	only via HCRU	n. r.
Vekeman et al. 2015 [35]	Retrospective, longitudinal cohort study, bottom-up	University Medical Center Utrecht (UMCU)/Netherlands	CKD stage/ AML size	kidney, lung	Diagnosis of TSC according to the revised 1998 TSC criteria	369^*^ (children and adults)	0	1990–2012	22	mean 14.3	32.4 (SD 23.7) CKD stage I	n. r.	n. r.	n. r.	n. r.	N/A	yes	yes	yes	yes	n. r.	n. r.	only via clinical information and HCRU	n. r.
Crall et al. 2016 [108]	Cross-sectional, bottom-up	web-based survey and survey of out-patients/USA	TSC with AF and caregivers	skin	Inclusion: clinical or genetic diagnosis of TSC, age > 6 years at enrollment /caregiver of a patient exclusion: uncertain diagnosis of TSC/ severe neurologic deficits/ survey noncompletion	219 (children and adults)	161	2014–2015	N/A	N/A	n. r.	n. r.	n. r.	94 (42.9)	n. r.	73.5	n. r.	yes	n. r.	n. r.	n. r.	n. r.	Children’s Dermatology Life Quality Index (CDLQI)	Childhood Atopic Dermatitis Impact Scale (CADIS)
Fallah et al. 2016 [109]	Cost-utility analysis, bottom-up and top-down	literature search (clinical data), Tufts CEA registry, Agency for Healthcare Research and Quality (AHRQ) Medical Expenditure Panel Survey/ USA	TSC + E only (model)	brain (seizures)	N/A	N/A	N/A	2000–2015	15	15 for clinical outcomes, cost data updated to 2016 USD	N/A	N/A	N/A	N/A	N/A (100)	N/A	n. r.	n. r.	yes	yes	n. r.	n. r.	n. r.	n. r.
Kingswood et al. 2016a [110]	Retrospective cohort study, bottom-up and top-down	National Health Service (NHS) databases CPRD (clinical practice research datalink) and CPRD-HES (hospital event statistics)/ UK	all TSC/ general population comparator	all	Diagnosis of TSC between 1 January 1987 and 30 June 2013 and at least 3 years of continuous data prior to the last available record	278^***^ (children and adults)	0^***^	1987–2013	26	at least 3	31.5 (SD 18.7)	n. r.	n. r.	147 (53)	n. r.	N/A	yes	yes	yes	yes	n. r.	n. r.	only via HCRU	n. r.
Kingswood et al. 2016b [111]	Retrospective cohort study, bottom-up and top-down	National Health Service (NHS) databases CPRD (clinical practice research datalink) and CPRD-HES (hospital event statistics)/ UK	TSC + renal manifestations/ general population comparator	kidney	See Kingswood et al. 2016a	79^***^ (children and adults)	0^***^	1987–2013	26	at least 3	38,3 (SD 17.1)	n. r.	n. r.	37 (46.7)	8 (11.3)	N/A	yes	yes	yes	yes	n. r.	n. r.	only via HCRU	n. r.
Kingswood et al. 2016c [42]	Retrospective cohort study, bottom-up and top-down	National Health Service (NHS) databases CPRD (clinical practice research datalink) and CPRD-HES (hospital event statistics)/ UK	all TSC/ general population comparator	all	Diagnosis of TSC between 1 January 1987 and 30 June 2013	334^***^ (children and adults)	0^***^	1987–2013	26	mean 17.4 (SD 6.4)	30.3 (SD 18.6)	n. r.	n. r.	177 (53)	n. r. (29)	N/A	n. r.	n. r.	n. r.	n. r.	n. r.	yes	only via mortality	n. r.
Wilson et al. 2016 [112]	Retrospective, top-down	Agency for Healthcare Research and Quality (AHRQ) National Inpatient Sample (NIS) database/ USA	all TSC	all	All TSC patients in the database	5655 (hospital admissions) (children and adults)	0	2000–2010	10	n. r.	22.3 (SD 19.5)	n. r.	n. r.	n. r. (52.5)	n. r.	N/A	yes	n. r.	yes	n. r.	n. r.	n. r.	only via HCRU	n. r.
Amin et al. 2017 [113]	Retrospective chart review, bottom-up	Bath TSC clinic database/ UK	all TSC	all	Definite diagnosis of TSC (International TSC Consensus Group)	284 (children and adults)	0	1981–2015	34	median 8 (IQR 3–17)	w/ ID 25 (IQR 15–36), wo/ ID 28 (IQR 17–43)	n. r.	n. r.	142 (50)	n. r.	N/A	n. r.	n. r.	n. r.	n. r.	n. r.	yes	only via mortality	n. r.
Shepherd et al. 2017 [23]	Retrospective cohort study, bottom-up and top-down	National Health Service (NHS) databases (EMR, HES, clinical practice research datalink)/ UK	all TSC/ TSC + E, TSC + E/ general population comparator	all	Recorded diagnosis of TSC in study period and at least 3 years of continuous data prior to the last available record	209 (children and adults)	0	1997–2012	15	at least 3	26.8 (SD 17.8)	N/A	N/A	102 (48.8)	81 (38.8)	N/A	yes	yes	yes	yes	n. r.	n. r.	only via clinical information and HCRU	n. r.
Song X. et al. 2017 [114]	Cross-sectional, top-down	Truven Commercial Claims and Medicaid database/ USA	TSC + AML/ controls in Commercial/ Medicaid databases	all	Patients diagnosed with TSC-renal AML in the databases	743 (children and adults)	0	2000–2013	13	37.0 (SD 31.1) to 87.2 (SD 42.9)	n. r.	36.9 (SD 13.0)	9.7 / 6.9 (SD 5.2)	n. r.	256 (34.5)	N/A	yes	yes	yes	n. r.	n. r.	n. r.	only via HCRU	n. r.
Welin et al. 2017 [27]	Retrospective, top-down	National Board of Health and Welfare (NBHW) national registers/ Sweden	all TSC	all	All patients with TSC diagnosis in national register	551 (children and adults)	0	2004–2014	10	mean 8.8	n. r.	n. r.	n. r.	339 (45.1)	238 (61.6) at first observation	N/A	yes	yes	n. r.	n. r.	n. r.	yes	only via HCRU	n. r.
Both et al. 2018 [115]	Qualitative study using semistructured interviews	Dutch Foundation for patients with TSC, healthcare providers/ Netherlands	TSC patients/ parents	all	Adolescent TSC patients 18–30 years old with a definitive TSC diagnosis; parents of children with TSC of 18 years, or older, or younger and having transitioned into adult care	28 (children and adults)	12	n. r.	n. r.	n. r.	R 17–30	n. r.	n. r.	n. r.	n. r.	n. r.	n. r.	n. r.	n. r.	n. r.	n. r.	n. r.	TSC-related themes, categorized by the ICF	TSC-related themes, categorized by the ICF
Hamer et al. 2018 [116]	Retrospective, multi-center cohort study (12), bottom-up	survey of patients from 12 German epilepsy centers/ Germany	all TSC	all	Patients aged ≥18 years with a confirmed TSC diagnosis according to clinical Gomez criteria and/or genetic testing	262 (only adults)	0	2010–2015	5	5	36.2 (SD 9)	36.2 (SD 9)	N/A	122 (46.6)	0	N/A	yes	yes	n. r.	n. r.	n. r.	n. r.	only via HCRU	n. r.
Kopp et al. 2018 [117]	Retrospective observational, bottom-up	Carol and James Herscot Center for Tuberous Sclerosis Complex, Massachusetts General Hospital (MGH)/ USA	all TSC	all	All patients under the age of 18 with TSC	99 (only children)	0	2001–2007	6	n. r.	N/A	7.7 (SD 4.2)	7.7 (SD 4.2)	54 (54.5)	99 (100)	N/A	n. r.	n. r.	n. r.	n. r.	n. r.	n. r.	n. r.	Symptom Checklist-90—Revised (SCL-90-R), Parenting Stress Index (Short Form)
Mowrey et al. 2018 [118]	Cross-sectional, bottom-up	web-based survey/ USA	all TSC	all	Having a diagnosis of TSC, age of 18 years or older, ability to independently complete a 30-min electronic survey.	71 (only adults)	0	2017–2018	N/A	3 months	43.7 (SD 13.2)	43.7 (SD 13.2)	N/A	52 (73)	0	N/A	yes	yes	n. r.	n. r.	n. r.	n. r.	Brief-Illness Perceptions Questionnaire (Brief-IPQ), Beck Anxiety Inventory (BAI), and Beck Depression Inventory-II (BDI-II).	n. r.
Rentz et al. 2018 [﻿119﻿]	Cross-sectional, non-interventional, bottom-up	web-based survey/USA	paediatric/ adult TSC + AML patients/ caregivers	kidney	see Rentz et al. 2015a	182^**^ (110 AML) (children and adults)	59^**^	2012	N/A	2 months (May and June 2012)	29.1 (SD 16.6)	36.4 (SD 13.6)	10.6 (SD 4.7)	n. r.	31 (28.2)	54	yes	yes	n. r.	n. r.	n. r.	n. r.	SF-12, HDI-SF	SF-12, HDI-SF
Skalicky et al. 2018 [﻿119﻿]	Cross-sectional, bottom-up	web-based survey/ USA	paediatric/ adult TSC patients/ caregivers	all	see Rentz et al. 2015a	609^**^ (children and adults)	275^**^	2012	N/A	2 months (May and June 2012)	28.9 (SD 18)	38 (SD 13.1)	7.1 (SD 4.6)	n. r.	179 (29.4)	45.2	yes	yes	yes	n. r.	yes	n. r.	WPAI CIQ-SHP version 2	WPAI CIQ-SHP version 2; out-of-pocket indirect healthcare spending related to time and money spent for TSC-related medical care travel and childcare expenses
Song J. et al. 2018 [120]	Retrospective, bottom-up and top-down	National Tuberous Sclerosis Association (NTSA), TSC Natural History Database/ USA, Belgium	TSC + E only	all	All TSC patients in the database	1110(children and adults)	0	2006–2014	8	4.3	n. r.	n. r.	n. r.	535 (48.2)	n. r.	N/A	yes	yes	n. r.	n. r.	n. r.	n. r.	only via HCRU	n. r.
Amin et al. 2019 [121]	Cross-sectional, non-interventional	Bath TS clinic/ UK	All TSC	all	TSC, as defined by the International Tuberous Sclerosis Complex Consensus Group	91	n. r.	2014	N/A	n. r.	n. r.	34	12	50%	35 (38.5)	n. r.	n. r.	n. r.	n. r.	n. r.	n. r.	n. r.	Pediatric Quality of Life Inventory (PedsQL) (children) SF-36 (adults)	n. r.
Bar et al. 2019 [53]	Cross-sectional, bottom-up	Written questionnaire/ network of the reference center for rare epilepsies and TSC (Necker-Enfants Malades and university hospitals of Saint-Etienne, Lille and Lyon) and the French association for TSC (ASTB), France	TSC patients in transition from pediatric care	all	Age > 18 years and confirmed diagnosis of TSC before the age of 16	60 (only adults)	30	2014	N/A	n. r.	32 (range 18–55)	32 (range 18–55)	N/A	29 (49%)	N/A	50	yes	yes	n.r.	n.r.	yes	n.r.	Quality of Life Scale (QOLS), Multidimensional Fatigue Inventory-20 (MFI-20)	n.r.
Tritton et al. 2019 [122]	Cross-sectional, bottom-up	Web-based survey/ USA and Europe	TSC + epilepsy	all	Clinical diagnosis of TSC and epilepsy	186	N/A	2017–2018	N/A	N/A	27.3	n. r.	n. r.	82 (44.1)	70 (37.6)	N/A	n. r.	n. r.	n. r.	n. r.	n. r.	n. r.	European quality of life (EQ – 5 dimensions – 3 levels)	n. r.
Vergeer et al. 2019 [123]	Single-center retrospective chart review, bottom-up	University Medical Center Utrecht (UMCU)/Netherlands	TSC with AML, SEGA and/or epilepsy	all	Diagnosis of TSC according to the revised 1998 TSC criteria	363^*^	0	1990–2015 (2012)	16	N/A	n. r.	n. r.	n. r.	n. r	n. r.	n. r.	n. r.	n. r.	n. r.	n. r.	n. r.	n. r.	Health Utility Index version 3 (HUI-3)	n. r.
Zak et al. 2019 [124]	Retrospective chart review, bottom-up	Cincinnati Children’s Hospital Medical Center TSC clinic/ USA	all TSC	all	All patients who attended the CCHMC TSC clinic during the study period, National Death Index (NDI)	567 (children and adults)	0	1998–2016	18	n. r.	n. r.	n. r.	n. r.	247 (> 18) (%n. r.)	n. r.	n. r.	n. r.	n. r.	n. r.	n. r.	n. r.	yes	only via mortality	n. r.

*, ** and *** denote studies with overlapping cohorts, *AF* facial angiofibroma, *AML* angiomyolipoma, *LAM* lymphangioleiomyomatosis, *R* range, *SD* standard deviation, *SEGA* subependymal giant cell astrocytoma, *TSC+E* TSC+epilepsy

#### Health care resource use

##### Outpatient/inpatient visits

All evaluated studies show that most TSC patients have a high rate of outpatient physician contacts, regardless of the medical system [23, 56] (see Table 3). Rates of physician contact generally are much higher than in the general population, three times as high in TSC patients in the UK [23]. However, in the UK, a high rate of adult general practitioner visits contrasted with an 88.5% rate of individuals who had never seen a neurologist and one-third of pediatric patients who had not seen a pediatrician during the last 3 years [23]. Data from Germany [116] revealed that one-half of people with TSC (51.5%) visited an epilepsy center less than once a year, and 46.6% scheduled at least two follow-up visits per year. In general, children were most frequently seen by neurologists, pediatricians, and ophthalmologists, adult patients most often visited neurologists, psychiatrists, and dermatologists, probably reflecting the changing clinical course of TSC throughout life [23]. In Swedish individuals with TSC [27], almost all (87.8%) of the study participants had experienced an outpatient visit with an International Classification of Disease 10th Edition (ICD-10) code identifying epilepsy.

**Table 3:** Health care resource use in TSC patients

Measure	Country of study origin	Value
Outpatient visits		
Overall contact with physician		
within the past year	USA [56]	99% of children and 98% of adults
within the past year for diagnosis “epilepsy”	Sweden [27]	87.8%
per year (neurologist)	Germany [116]	42.0%
Frequency of physician contact		
within the past year (overall)	USA [56]	22 (on average almost two times a month)
per year (overall)	Canada [104] Sweden [27]	14 (mean, SD: 1.0), significantly more than the general population (8.3; SD: 0.3) 4.70 (mean, SD: 4.17); 1.65 (mean, SD: 1.95) for the ICD-10 code “epilepsy”
per year (outpatient specialist)	Canada [104]	8.7 (mean, SD 0.6)
within the past 3 years (general practitioner)	UK [23]	60.8 (on average)
within the past 3 years (outpatient specialist)	UK [23]	15.3 (on average)
Inpatient visits		
Overall rate of hospital admission		
within 1 year	USA [56]	37%
within 5 years	USA [24]	85%
within 16 years	Canada [104]	84.8%
within 5 years (intensive care unit admission)	USA [24]	22.1%
Frequency of hospital admission		
within one year	USA [24]	0.5 (0.28 for neurological complications)
within the past year (emergency room)	USA [56]	2 (on average)
within the past year (excluding emergency room)	USA [56]	2 (on average)
within the past 3 years	UK [23]	3.4 (on average; two [23] to three [111] times the general population)
per 10 person-years	Canada [104]	2.5; SD: 3.2 (vs. 1.3 admissions; SD: 1.5 in the general population) 5.8; SD: 2.1 for TSC-LAM
Annual length of stay	USA [56] Sweden [27]	5.4 days (mean, SD: 3.0) 3.25 days (mean, SD: 5.61) overall, 2.06 days (mean, SD 4.50) due to epilepsy
Average length of stay	USA [24]	6.2 days (on average; 6 days for admissions due to neurological complications)
Diagnostic procedures		
Number of patients with three or more procedures/year	USA [24]	90.5%
Average number of procedures/year	USA [56]	9
Patients with (at least one)		
EEG/year	USA [24]	93.7%
EEG/year	UK [23]	46.9% of children 10.9% of adults.
Long-term EEG/year	USA [24]	64%
MRI/year	USA [24]	90.5%
MRI/year	USA [56]	66%
MRI/year	UK [23]	58.0% of children 21.1% of adults
MRI/ last 3 years	Germany [116]	78.6%
Regular MRI in SEGA	France [53]	15%
CT/year	USA [24]	55.8%
Blood test/year	USA [56]	57%
Ultrasound/year	USA [56]	45%
Ophthalmologic evaluation/year	USA [56]	40%
Renal screening/ last 3 years	Germany [116]	56.1% (specific screening modality not reported)
Renal screening	France [53]	78, 40% regularly every 2 years
Psychiatric evaluation in those with TAND	France [53]	13% (psychological or psychiatric follow-up)
ASD and other medication use		
mTOR inhibitor	Sweden [27]	15.3% (for any indication; not differentiated)
ASD use		
in individuals with epilepsy	Sweden [27]	97.9% (378/386)
in children	USA [56]	69%
in adults	USA [56] UK [23]	25% 88%
Most common ASD	Sweden [27]	valproate (174/386; 45.1%) lamotrigine (167/386; 43.3%) carbamazepine (145/386; 37.6%) levetiracetam (141/386; 36.5%)
Most common ASD	UK [23, 110]	Carbamazepine (48.8%) Valproate (48.8%) Vigabatrine (43.2% children vs. 24.4% adults)
Anxiolytic medication use		
overall	Sweden [27]	72.5% (includes the potential use of benzodiazepines as ASD; not differentiated)
in children	USA [56]	21%
in adults	USA [56]	37%
Antipsychotic medication use		
overall	Sweden [27]	16.6%
Most common antipsychotic medication	Sweden [27]	risperidone (11.4%)
Anxiolytic medication use		
in children/ past 3 years	UK [23, 110]	20.3–37% (includes hypnotic medication)
in adults/ past 3 years	UK [110]	33.3% (includes hypnotic medication)
Antidepressants in children	USA [56]	15%
Psychoanaleptic medication use		
overall	Sweden [27]	23.6%
in children	USA [23]	19.9%
in adults	USA [56]	20%
Most common	Sweden [27]	methylphenidate (7.3%)
Surgical procedures		
Epilepsy surgery	USA/Belgium [120], Germany [126], Sweden [27], multinational [26]	6.5–25.3%
Surgery for SEGA		
Brain surgery (no differentiation)	USA, UK [22, 23]	7.2–8.4%
Craniotomy	USA [112]	5%
Cerebral shunt	USA [112]	3.5%
Vagal nerve stimulator implantation	Multinational [26], Sweden [27]	3.8–6.0%

*ASD* antiseizure drug, *EEG* electroencephalogram, *CT* computed tomography, *ICD-10* International Classification of Diseases - 10th Revision, *MRI* magnetic resonance imaging, *SD* standard deviation, *SEGA* subependymal giant cell astrocytoma, *TAND* TSC-associated neuropsychiatric disorders, *TSC* tuberous sclerosis complex, *TSC-LAM* tuberous sclerosis complex with lymphangioleiomyomatosis

Individuals with TSC-associated epilepsy living in the UK had on average 3.4 inpatient admissions in three years, which is almost three times the rate of the general population [23] with similar to slightly lower numbers reported from the general TSC population in the US [56] and Canada [104]. Mean annual length of stay (LOS) was 5.4 days (SD: 3.0) in the US study [56], longer than in a Swedish study (mean 3.25 days; SD: 5.61) [27]. More than half (59.8%) of individuals had an inpatient visit with an ICD-10 code identifying epilepsy in the Swedish study [27]. Another study from the US reported about half of hospitalizations per year per patient were due to neurological complications. In a US study, persons with TSC-associated neurological manifestations required significantly more hospitalizations than did those without. Children with TSC and developmental impairment had significantly more ICU stays as compared with cognitively unimpaired children [24]. Individuals with TSC and epileptic spasms or refractory epilepsy and young patients had the highest mean health care utilization in a Swedish study [27]. This pattern was true for all evaluated categories of health care utilization [27].

##### Diagnostic procedures

Individuals with TSC receive a high number of diagnostic procedures each year. Consistently, the most commonly performed procedures in individuals with TSC are EEG (10.9–93.7%/year), MRI for any indication (22.1–90.5%), CT for any indication (55.8%), blood tests (57%) and ultrasound (45%) [56]. However, the frequency varies between different medical systems. A study from the US [24] found that, within 5 years, 90.5% underwent three or more diagnostic procedures. The frequency of diagnostic test in a UK study [23] was much lower (on average 1.1/3 years), which was still 5 times more than in the general population. In a Dutch study, individuals with TSC and CKD stage III had more scans, nonsurgical procedures, and specialist visits than did those with lower stages of CKD [35]. A multicenter survey from Germany [116] reported that presurgical diagnostics were performed in 27% of patients. Of note, in 34% of individuals with TSC and epilepsy in the UK study, no diagnostic test had ever been performed, while 24.9% had only ever had one test (see Table 3). Several studies show an incomplete observation of the TSC Surveillance and Management Recommendations [127] regarding diagnostic renal screening. In a German study, 56.1% of individuals had had renal screening (modality not reported) in the last 3 years [116] and a psychiatric evaluation had been performed in only 13% of individuals with TAND in a French study [53]. Regular neurologic follow-up (62%), regular SEGA brain imaging (15%) and nephrologic screening (40%) also was not regularly applied in all patients [53]. In an Australian cohort, adults were significantly less likely to follow surveillance guidelines as compared with children (36% vs. 89%) [128].

##### Anti-seizure drugs (ASDs) and other medication use

Data from several countries show that ASD are the most common drug class used in individuals with TSC (69–97.9%), followed by anxiolytic medication (21–72.5%), psychoanaleptics (19.9–23.6%) and antipsychotics (16.6–37%%) [27]. Inhibitors of mTOR were used in 15.3% in one study, without differentiation of application or indication [27]. Anxiolytic therapy may be overstated, as benzodiazepines are also used as ASD and several studies do not precisely differentiate between related drug classes or indications [23, 27, 56, 110]. In TOSCA, 98.1% of patients with focal seizures received ASD treatment, most commonly with GABAergic anticonvulsants (66%) [26]. In the Swedish national insurance database [27] the most common ASD were valproate (45.1%), lamotrigine (43.3%), carbamazepine (37.6%), and levetiracetam (36.5%). Vigabatrine is used significantly more often by children (43.2%) than adults (24.4%) as reported from UK data [23]. Several studies showed that individuals with TSC require between four and eight times more prescriptions than the general population [23, 26, 110]. In a UK study, hypnotics or antipsychotic drugs (not differentiated) were prescribed twice as often in individuals with TSC than in the general population. In particular, the difference was sevenfold in children with TSC as compared with in the general population [110]. However, in a French study in which 80% had intellectual disabilities and 70% had psychiatric disorders, only 20% received pharmacotherapy [53]. Overall medication use in individuals with TSC and renal manifestation was found to be higher in CKD stage III than in lower stages [35]. The ketogenic diet was used by 1.6 to 4.7% of individuals [26, 27].

##### Other procedures (surgery, etc.)

A study examining [120] US and Belgian TSC patients reported a rate of epilepsy surgery of 25.3%, a German study of 9% [126] while national insurance data offered a rate of 6.5% for Sweden [27], similar to the 6.9% of epilepsy patients in TOSCA [26]. Brain surgery without further differentiation regarding indication was reported in 8.4% in a US study [24] and 7.2% in a UK study [23]. In a US database analysis of 5655 individuals with TSC, 5% had received a craniotomy and 3.5% had a cerebral shunt. The median LOS for these procedures was 3 days [interquartile range (IQR): 2–6 days] [112]. Additionally, in a US study [24], brain electrodes were implanted in 6.3% of patients, but the rationale (diagnostic vs. therapeutic) was not provided in detail. Reported rates of VNS implantation are between 3.8% in TOSCA and 6.0% [26, 27]. In a small study, nine of 11 patients (82%) had at least a 67% reduction in seizure burden [129]. Seizure freedom after epilepsy surgery was reported as 57% in a German study [116], in line with smaller studies performed on children [126, 130], and a systematic review (Engel class 1 achieved in 57% of TSC patients) [131]. Intellectual ability was significantly better in those with Engel class 1 outcome. Another study on outcomes of pediatric epilepsy surgery found at least a moderate improvement was achieved in 46 to 85% of patients. There was a significant correlation between quality of life measures and a favorable Engel outcome class [132]. For a review on epilepsy surgery in TSC, see Jansen et al. and Evans et al. [131, 133].

Regarding kidney interventions, individuals with TSC-associated renal impairment and CKD stage III underwent more surgeries than did those in lower stages [35]. Eijkemans et al. [86] noted that individuals in the same Dutch cohort with higher stages of AML required more renal embolization. Renal transplantation does rarely occur in TSC and generally has favorable results. The BOI of this surgical intervention has not been evaluated [88, 89].

##### Other therapies (physical, educational, etc.)

Only very few studies have looked at therapies in TSC that are not administered by physicians. In a US study [24], almost half of patients (43.2%) required rehabilitation services, including most commonly occupational and speech-language therapy (each 34.7%). Physical therapy was performed in 31.6% and special education services in 14.7% of patients, respectively. Developmentally impaired children and individuals with neurological manifestation in general required more rehabilitative effort than did those without. The low number of special education services reported in this study is probably due to incomplete assessment in some age groups [24]. In a French cohort with a prevalence of psychiatric disorders of 70%, only 13% had a psychological or psychiatric follow-up [53].

##### TSC centers

The share of patients treated at TSC centers as opposed to non-integrated care was reported by 10 studies. In those not recruiting patients from a TSC center, the rate of patients treated at a TSC center was between 27.9 and 51.7%. Data from the US suggested that almost half of all patients received their care at TSC centers [56]. A German multicenter survey [116] also showed that medical care involved a TSC center in 27.9% of cases, and 36.6% of patients reported the visit of an urologist or nephrologist in addition to the epilepsy center consultation.

#### Direct costs

A UK study [110] estimated that the total costs incurred by individuals with TSC were 2.7 times higher than such in the general UK population. An individual with TSC reportedly incurs a mean total cost of GBP 12,681 (PPP-$ 17,629) over a three-year period as compared with GBP 4777 (PPP-$ 6641) per general population patient. On average, the highest per-patient costs were incurred by (overall rare) respiratory manifestations (GBP 40,312, PPP-$ 56,040). Structural brain manifestations led to the highest three-year cost (GBP 22,139, PPP-$ 30,777), followed by renal and urinary tract manifestations (GBP 15,162, PPP-$ 21,078) and nervous system manifestations (GBP 14,355, PPP-$ 19,956). Manifestations in the renal and nervous system were each found to significantly impact costs. Also, the number of organ systems involved was found to be a significant cost driver, with statistical significance persisting as the number of manifestations increased. However, age and sex were not found to significantly impact costs [110].

In a subgroup analysis, the same UK group [111] estimated the direct costs of TSC patients with renal manifestations. The total average cost for a TSC patient with renal manifestations was almost three times higher than that in the general population (GBP 15,162, PPP-$ 21,078 vs. GBP 5672, PPP-$ 7885) in 2014. All cost aspects were substantially higher in individuals with TSC-associated renal manifestations. Among TSC patients, a more than twofold increase in direct costs was seen for GP visits and inpatient hospitalizations, while more than three times the typical cost was accrued for outpatient visits and primary care drugs (see Table 4). At the same time, no kidney-related procedures were performed in 70.9% of individuals with TSC, while more than one-quarter did not undergo the recommended amount of renal screening procedures. A Dutch study [35] reported that higher health-care resource use (HRCU) is associated with male gender, CKD greater than stage I, AML size of 3.5 cm or larger, embolization, and the presence of moderate or severe LAM. Higher costs in CKD stage V were consequently induced by dialysis. The overall costs were EUR 1275 (PPP-$ 1715) for CKD stage I, EUR 3547 (PPP-$ 4770) for stage IV, and EUR 31,916 (PPP-$ 42,921) for stage V (defined as any patient requiring dialysis), respectively (all costs originally represented in 2012 EUR). The single biggest cost in CKD stages I and II was surgery. Conversely, for stages III and IV, it was medication and, for stage V, costs were primarily driven by dialysis. Patients aged 60 years or older had lower costs as compared with patients aged younger than 20 years, maybe owing to less frequent testing. In a US study based on commercial and governmental claims data [114] adult and pediatric TSC patients with AML utilized more resources than did the general population. Direct health care costs (in 2013 USD) in commercial claims were between USD 29,240 (PPP-$ 31,605) and USD 48,499 (PPP-$ 52,422) for TSC patients, or 14 to 22 times higher than that in the general population.

TSC patients with LAM also have significantly higher health care costs. In a Canadian study, health care costs in the TSC population (1004 individuals) were almost twice as high as in the general population. In addition, 38 patients with LAM had even higher health care costs [104] (see Table 4).

**Table 4:** Direct costs in TSC patients

Study	N patients total	Group	Costing year	Original cost figure given	Cost/year/patient (calculated)	Cost in 2018 PPP-$	Significant cost drivers (in regression analysis)	Out-of-pocket spending	Cost per admission	Further measures
Shepherd et al. 2017 [23]	286	TSC and epilepsy	2014	GBP 14,335 / 3y	GBP 4778	6643	number of organ systems involved; combination of kidney and urinary/dermatological manifestations	n. r.	n. r.	
Vekeman et al. 2015 – CKD Stage I [35]	369	TSC and renal manifestations	2012	EUR 1275	EUR 1275	1715	CKD stage V vs. CKD stage I; AML size > = 3,5 cm; comorbid moderate or severe LAM	n.r.	n. r.	
Vekeman et al. 2015 – CKD Stage V [35]			2012	EUR 30,641	EUR 30,641	41,207				
Kingswood et al. 2016a [110]	278^b^	All TSC	2014	GBP 12,681 /3y	GBP 4227	5876	number of organ systems involved; kidney and urinary tract manifestations, nervous system manifestations; *pairwise:* circulatory/kidney and urinary tract, nervous system/psychiatric, dermatological/kidney & urinary tract manifestations	n. r.	n. r.	*GP visits* (GBP 3433, PPP-$ 4772 vs. GBP 1283, PPP-$ 1784) *inpatient hospitalizations* (GBP 7050, PPP-$ 9801 vs. GBP 3298, PPP-$ 4585) *outpatient visits* (GBP 2071, PPP-$ 2879 vs. GBP 613, PPP-$ 852) *primary care drugs* (GBP 2607, PPP-$ 3624 vs. GBP 479, PPP-$ 666)
Kingswood et al. 2016b [111]	79^b^	TSC and renal manifestations	2014	GBP 15,162 / 3y	GBP 5054	7026	number of primary TSC manifestations; nervous system manifestations; *pairwise:* combination of dermatology/psychiatric manifestations	n. r.	n. r.	
Skalicky et al. 2018 [119]	609^a^	all TSC	2018	Tests/procedures: USD 5499-20,403 Hospital expenses: USD 1263-5533 Doctor’s visits: USD 1646-4462 ER visits: USD 702–2671	n. r.	n. r.	n. r.	For tests and/or procedures: USD 5499-20,403 Hospital expenses: USD 1263-5533 Doctor’s visits: USD 1646-4462 ER visits: USD 702–2671	n. r.	
Skalicky et al. 2015 [106]	116^a^	TSC and SEGA	2015	USD 80–129	n. r.	n. r.	n. r.	median monthly: USD 80–129 (about 50% of patients)	n.r.	
Song X. et al. 2017 [114]	743	TSC and AML	2013	USD 29,240-48,499	USD 29,240 to 48,499	31,605 to 52,422	n. r.	n. r.	n. r.	
Wilson et al. 2016 [112]	5655 (admissions)	all TSC	n. r.	USD 14,807 (IQR 7319-31,180)	n. r.	n. r.	n. r.	n. r.	USD 14,807 (IQR 7319-31,180)	
Sun et al. 2015 [107]	47	TSC and SEGA surgery	2010	USD 8543.1 (SD 11,187.6) for presurgical year USD 85,397 (SD 56,258.9) for postsurgical year	USD 8543 to 85,397	9805 to 98,008	n. r.	n. r.	n. r.	Presurgical year: *inpatient* USD 3770, PPP-$ 4327 *outpatient treatments* USD 3473, PPP-$ 3986 *Medications* USD 1300, PPP-$ 1492
										postsurgical year *surgery* USD 71,562, PPP-$ 82,130 *Outpatient costs* USD 11,497 PPP-$ 13,195 *medication costs* USD 2338, PPP-$ 2683 (costs 1.6 to 4.3 times higher than in the presurgical year [inpatient: 4.3:1, outpatient: 2.5:1, medication: 1.6:1, and total: 3.1:1])
Kristof et al. 2015 [104]	1004 (TSC) 29 (LAM)	TSC and/or LAM	2011	TSC: CAD 513 (SD 5.83) LAM: CAD 1434 (SD 10.14) TSC-LAM: CAD 1718 (SD 10.53) General population control: CAD 281 (SD 4.37)	CAD 513 to 1718	424 to 1421	n. r.	n. r.	n. r.	

^a^, ^b^ denotes patients originating from same cohort; *AML* Angiomyolipoma, *CAD Canadian dollar, CCHMC* *Cincinnati Children’s Hospital Medical Center, CKD Chronic kidney disease, ER* Emergency room, *GBP Great Britain pound, HDI-SF* Hamilton depression inventory short form, *ICF* International classification of functioning, disability, and health, *IQR* Interquartile range, *LAM* Lymphangioleiomyomatosis, *n. r.* Not reported, *PedsQL Pediatric quality of life inventory, PPPY* Cost per person per year, *PPP-$* International Dollar purchasing power parities, *SEGA* Subependymal giant astrocytoma, *SF12* Short form health survey 12-item, version 2, *TSC* Tuberous sclerosis complex, *USD United States dollar, WPAI CIQ-SHP* Work Productivity and Activity Impairment plus Classroom Impairment Questions: Special Health Problem (WPAI CIQ-SHP), version 2 questionnaire; for data sources please refer to Table 2

Sun et al. [107] evaluated costs in patients undergoing SEGA resective surgery. In the postsurgical year, patient costs were three-fold higher than in the presurgical year, with a high inpatient proportion being attributed to surgery. Long-term costs were not assessed by the study. Another US study [112] showed that median hospital stay charges for TSC patients with craniotomy were USD 65,885 (IQR: USD 39,195–120,180). This was more than four times the financial amount charged of those not receiving craniotomy. Long-term follow-up costs were likewise not assessed in the study.

Skalicky et al. [119] analyzed economic burden in a cohort described previously [56]. Adult patients had significantly higher out-of-pocket direct costs than did pediatric patients. In this study, more than two-thirds of patients worked for pay, but the type (primary vs. subsidized labor market) was not stated by the authors. TSC patients had substantial yearly out-of-pocket costs (median of USD 1750 for pediatric and median of USD 3270 for adult patients, respectively) for both outpatient and hospital care in a US cohort [106]. In a study on BOI of facial angiofibromas in a US population, the cost of medication and lack of a suitable pharmacy were seen as biggest hurdles in receiving topical rapamycin therapy [108], but average costs were not given.

#### Projected costs

Fallah et al. [109] estimated the theoretical cost-effectiveness of four different therapy strategies in pediatric TSC patients with drug-refractory epilepsy, specifically epilepsy surgery, VNS, ketogenic diet, and carbamazepine as an additional third ASD. The cost-effectiveness was modeled based on adjusted historic costs and data from an open cost-effectiveness registry. In pediatric patients with drug refractoriness to two ASDs and the fundamental possibility of epilepsy surgery, the addition of a third ASD was the most cost-effective solution (USD 6568 for 4.14 QALY). In a further estimate, patients with three ASDs who did not achieve seizure freedom could most cost-effectively be helped by epilepsy surgery (USD 77,675 for 4.38 QALY), followed by the addition of a fourth ASD (USD 50,862 for 4.11 QALY) and ketogenic diet treatment (USD 16,228 for 3.60 QALY). Which of those strategies was best depended upon health-care system resources. In resource-rich countries, epilepsy surgery was deemed as the most effective treatment and ketogenic diet in resource-limited environments. Mechanistic target of rapamycin (mTOR) inhibitor treatment for epilepsy alone was not a cost-effective treatment strategy based on the costs induced by the mTOR therapy in the historic reports, given at USD 134,436/year (range USD 142,737–160,462) [109].

#### Individual and caregiver burden of illness and quality of life

In comparison with the studies focusing on health care resource use, there are fewer studies focusing on individual BOI in TSC. In a UK study, impaired QoL in psychosocial and physical domains was apparent in all adults and children, regardless of the presence of epilepsy or intellectual disability [121] (as measured by the Pediatric Quality of Life Inventory [PedsQL] and the Short Form (36 items) Health Survey 36 [SF-36]). Nevertheless, quality of life is lower in those with TSC and epilepsy than in those with TSC with only renal AML, primarily attributable to reduced cognitive functioning [123]. Quality of life and daily functioning worsens with increasing seizure frequency or severity [122], and pharmacoresistant epilepsy significantly reduces QoL [121]. Older age and reduced daily functioning also negatively affect quality of life (as measured by the Health Utility Index version 3 [HUI-3] questionnaire) [123]. In a US web-based survey study [105], adults with TSC named skin lesions (15%), sleep problems (10%), and kidney complications (9%) as the “most bothersome” aspects of TSC. Crall et al. [108] showed that individuals with TSC experienced no negative impact of facial angiofibroma on their QoL, as measured by dermatological QoL scales. However, patients who received therapy for their AF reported better dermatological QoL than did those who did not. Quality of life in children with TSC is worse than in diabetes, cancer and inflammatory bowel disease, when evaluating the PedsQL [121]. Individuals with TSC patients had a better QoL than Alzheimer’s disease sufferers but worse than rheumatoid arthritis sufferers and the general healthy population in the HUI-3 [123].

One study reported on QoL measures as secondary treatment outcome of everolimus for children with pharmacoresistant epilepsy. After 12 weeks of treatment, the overall QoL was significantly better, driven by many domains (primarily by attention, behavior, other cognitive, social interaction, stigma, physical restrictions and social activity), as measured by the QOLCE [103]. In a study on resective and disconnective surgeries in pharmacoresistant epilepsy, the QoL showed significant improvement in all patients, especially patients with low preoperative intelligence quotient (IQ) and postoperative seizure freedom or disconnection of the corpus callosum, as measured by neuropsychological evaluation and the QOLIE-31 [101]. Of note, antiseizure medication and mTOR therapy can have adverse reactions, most commonly dizziness and nausea in ASD. Everolimus therapy leads to stomatitis in a substantative share of patients (43.2%, according to final results from the EXIST-1 [134], but rarely grade 3 or 4) and can increase the risk of pneumonia. The effects on BOI by these adverse reactions have not been studied explicitly.

A subgroup-analysis [56, 105] examined the physical and mental health burdens on caregivers providing assistance to individuals with TSC. Caregivers declared seizures (32%), cognitive impairments (25%), and skin lesions (15%) as the “most bothersome” concerns. Overall, caregivers of people with TSC had significantly lower QoL scores in both physical and mental domains and had more depressive symptoms than the general healthy US population [105]. Caregiver QoL is negatively affected by facial angiofibroma of the patient [108]. Behavioral problems, persisting seizures, and psychiatric comorbidities significantly increase parental stress [117]. In a qualitative study [115] in 16 individuals with TSC and 12 parents, the main concerns were mental and physical health, social participation, self-management skills, family planning, and the side effects of medications. Patients wished for multidisciplinary care that focused on the wellbeing of whole patient, including their family. At the same time, caregivers often feel overwhelmed and feel a lack of psychosocial support and orientation, as seen in a qualitative Italian study [102]. This study highlighted that many caregivers find support in patient organizations rather than in their extended personal network [102]. Transition from pediatric to adult health care frequently exacerbates the BOI in TSC patients due to changing health care providers and a loss of integrated care. A French study evaluated patient experiences during transition [53]. In comparison, pediatric care was more regular and multidisciplinary than adult care. Epilepsy followed by renal issues had the best transition (best rate of follow-up). For psychiatric and behavioural disorders, transition was worse. Notably, only half of patients with a normal intellectual development had clear knowledge about their disorder and the need for a regular monitoring. The most stressful part of transition was the change of care structure and/or caregivers. Of note, only 10 % of individuals in the study rated their quality of life as good or excellent while more than half rated it as mediocre or bad (18%) [53].

A US study [105] reported that caregivers and TSC patients both missed about one-tenth of work time due to the disorder (11 and 15%, respectively). Adults with TSC, however, reported less overall work productivity and felt more work time was impaired by the disorder. However, none of the studies provided indirect cost estimates. In the French study, a third of patients had a stable income, but in 65% salary was below the national minimum wage [53]. In a multinational study, only 17.7% reported working at least part-time (sector not reported) [122].

#### Mortality

Mortality is significantly higher in individuals with TSC than in the general population, please refer to Table 5 for details. A Dutch study demonstrated a fivefold higher mortality rate than that in the age- and gender-matched general population. In this study [86], within 15.8 years, 29 of 351 individuals with TSC died (standardized mortality ratio: 4.8; 95% confidence interval: 3.4–6.9). A separate Swedish study [27] found that 7.8% of individuals with TSC (*n* = 30) died during the study period (mean duration of observation: 8.82 years). In 50% (*n* = 15), death was directly related to TSC. A US study [113] retrospectively identified 284 patients who attended a single center between 1981 and 2015. At the time of research, 16 individuals (5.6%) had died from complications of TSC, and the median age at death was 33 years. Shepherd and Gomez [135] found in a US cohort that 48 of 355 individuals (13.5%) with TSC died. In addition, mortality in those with intellectual disabilities exceeds the mortality in those without [86, 113]. In one study, LAM shortened the life expectancy by 7 years in a US collective of women with TSC (70.5 vs. 63 years) [124].

**Table 5:** Studies reporting mortality in TSC patients

Study / cause of death	Epilepsy	Kidney	Brain structural	LAM	Other pulmonal	Infection/sepsis	Cardio-vascular	Unknown	Not described	Undiscriminated tumor / cancer	Undiscriminated, but TSC-associated	Certainly not TSC	Multiple causes	N of deceased patients in study	N of patients in study	Mortality (%)	Start evaluation period	End evaluation period	Calculated duration of evaluation period	Actual evaluated period
Welin et al. 2017 [27]	3	–	–	–	–	–	–	5	15	7	–	–	–	30	386	7.8	2004	2014	10	8.8
Amin et al. 2017 [113]	4	8	1	2		–	–	–	–	1	–	–	–	16	284	5.6	1981	2015	34	8
Shepherd et al. 2017 [135]	9	9	9	4		4	2			3	–	9		49	355	13.8	n. r.	n. r.	n. r.	n. r.
Eijkemans et al. 2015 [86]	3	9						13		4	–	–	–	29	351	8.3	1990	2012	22	15.8
Kingswood et al. 2016 [42]	–	–	–	–	–	–	–	–	16		–	–	–	16	334	4.8	1987	2013	26	17.4
Kristof et al. 2015 [104]	–	4	–	–	–	24	16		73	–	10	4		131	1004	13.0	1996	2011	15	n. r.
Zak et al. 2019 [124]	9	1	1	6	2	1	0	1	0	0		1	1	23	662	3.5	1998	2016	18	n. r.
**SUM**	28	31	11	12	2	29	18	19	104	15	10	14	1	**294**	**3376**	**Mean 8.7**	–	–	**Mean 20.8**	**Mean 12.5**

*LAM* Lymphangioleiomyomatosis, *n. r.* Not reported, *TSC* Tuberous sclerosis complex; for data sources please refer to Table 2

When aggregating the data on all published mortality numbers in individuals with TSC, the most common causes of TSC-attributable deaths are epilepsy [especially status epilepticus and sudden unexpected death in epilepsy (SUDEP)], kidney complications, and complications from infections [27, 42, 86, 104, 113, 124, 135]. Structural brain manifestations and LAM in women are other prevalent causes of TSC-associated death [100, 124]. While malignancies were commonly reported as a cause of death in the evaluated patients, further specifics were lacking. In general, the overall cancer rates are not elevated in TSC patients [136].

## Discussion

The individual burden of illness in TSC is highly variable, resulting from the individual’s particular clinical manifestation in multiple organ systems over time [137]. In general, TSC patients have a significantly increased BOI as compared with the general population. BOI is also higher than in many other chronic diseases [121, 123]. As evident by the results of this review, where 14 studies on quality of life used more than as many different questionnaires, standardized assessment of BOI in TSC is difficult. To facilitate BOI research in TSC, the use of evaluated and standardized questionnaires should be encouraged. While this is challenging in rare diseases, first steps in this direction have been made [99].

### Health care resource use

Independent of specific health care systems, hospitalization rates of individuals with TSC are at least twice as high as those in the general population. Children with cognitive impairment and severe forms of epilepsy have an especially high hospitalization risk and are more likely to require ICU treatment. Outpatient physician visits are even more frequent relative to the general population, which is plausibly explained by the high number of specialist that individuals with TSC need to see. However, there is a dearth of information regarding frequency of non-physician outpatient therapies for TSC. While many individuals with TSC and caregivers lament the lack of support in the domains of psychological and physical functioning [102], surprisingly little has been published regarding frequency of neuropsychological, occupational or physical therapy.

Not surprisingly, individuals with TSC also require more medication than the general population and a high number of anxiolytic and antipsychotic medication was reported from several health systems [27, 56]. A closer evaluation of these therapies seems necessary especially considering the insufficient diagnosis of TAND in many patients. The use of mTOR inhibitors might prevent epileptogenesis and some of the late organ manifestations in patients with TSC and has the potential to decrease HRCU in the long-term [138, 139], but this has not yet been investigated.

Individuals with TSC also undergo more diagnostic procedures than the general population. Nevertheless, studies have hinted at insufficient adherence to surveillance standards, especially in adults [128], and resulting ineffective compensation, e.g., through frequent GP visits [23]. Notably, a French study found that only 50% of adult patients without cognitive impairment were aware of the need for regular check-ups in TSC [53]. This could be alleviated by the improvement of the transition from integrated pediatric to the commonly fragmented adult health care sector. Transition seems to be especially problematic regarding psychiatric problems [53]. In all patients, transition should be guided towards specialized integrated TSC centers. This is especially true for those who are more severely affected, in whom regular screening (e.g. MRI) may be more difficult.

### Direct costs

TSC patients incur higher costs than the general population due to the chronic and multisystem nature of their disorder. In general, costs are at least twice as high as in the general population. These higher direct costs are due to variety of reasons, among them being higher in- and outpatient care use and the involvement of complicated medical operations such as brain surgery and renal procedures. Costs rise with the number of affected organ systems. Pulmonary complications from LAM can also result in substantial costs, although these complications are rare overall. Integrated care at TSC centers is a plausible strategy to reduce costs by eliminating wasteful diagnostics and reducing complications of TSC, but no data exist yet to support this notion. Data from a UK study [23] suggest that the loss of multidisciplinary care, which often occurs during transition, significantly reduces the quality and efficiency of medical care. To date, there is a dearth of studies directly evaluating the potential cost benefit and improvement in terms of HRCU of centralized care at TSC clinics. While 10 studies mentioned the share of patients treated at a TSC center, no explicit outcome in differences were reported. Especially, the benefits in the long-term treatment have not been adequately assessed in the presently available studies. This is equally true for studies focusing on the cost of neurosurgical interventions. One study calculating projected costs found that epilepsy surgery is a cost-effective treatment option in high-resource environments [109].

### Individual and caregiver quality of life

While it is difficult to assess an individual subjective BOI or to directly compare BOI from different organ manifestations ins TSC, neurological and psychiatric manifestations play an important role for individuals with TSC and their caregivers. Quality of life was significantly worse for those with epilepsy than with only renal AML in one study [121]. Quality of life is severely affected by lower cognitive functioning and pharmacoresistant epilepsy [121–123]. Everolimus has recently been approved as a specific disease-modifying drug in TSC and first results are encouraging regarding the reduction of BOI in some indications [103]. The same is true for epilepsy surgery [101]. Multimodal approaches should be used to identify more pharmacoresistant epilepsy candidates for surgery.

Caregivers are particularly burdened by caring for individuals affected by more severe forms of epilepsy such as West syndrome and correlating neurological and psychiatric manifestations [105, 117]. Against this background, it is surprising that, according to the results of the TOSCA study, neuropsychiatric symptoms in TSC patients remain incompletely assessed. The use of the TAND checklist developed by de Vries et al. [82] can possibly remedy this situation and lead to a better psychological and psychiatric care of the affected TSC patients. Caregivers seems to miss a high amount of work time, but unfortunately, data on this is sparse. Indirect costs should be a focus of further research. Caregiver burden should be openly discussed in an appropriate setting and help could be offered, e.g., by identifying and closing gaps in psychosocial support. Referral to patient advocacy groups may also be appropriate in many cases.

While it may be obvious to the practitioner that more severe manifestations of TSC, especially in the neurological and psychiatric domains, are severely stressful for the patient and his caregivers, there could be a discrepancy between the priority of symptoms for the individual and the external medical perspective regarding other manifestations. Skin lesions may not be seen as particularly grave but were among the most bothersome signs for adults with TSC in one study. Consequently, the treatment of facial angiofibroma improved QoL [108]. Thus, practitioners should openly discuss skin manifestations with the patient and refer them to appropriate dermatological care.

Mortality is significantly increased in TSC patients. When aggregating all published mortality data in TSC patients, we found that the most common causes of death were SUDEP, kidney complications, and complications from systemic infections. The high rate of SUDEP deaths may be explained by a high rate of drug-refractory epilepsy, which is a major risk factor of SUDEP. The risk of SUDEP should be discussed with patients with epilepsy and their caregivers in an appropriate setting [140, 141]. Infections such as aspiration pneumonia common in one study [104] can be sequelae of bilateral tonic-clonic seizures.

## Conclusions

Individuals with TSC and their caregivers share a high burden of disease, which is higher than in many other chronic diseases. Quality of life is reduced especially in those with pharmacoresistant epilepsy and reduced cognitive functioning. While individuals with TSC require a considerable amount of medical care, gaps in screening and treatment are apparent, especially regarding the treatment of TSC-associated neuropsychological disorders. Recent advancements in targeted therapy by mTOR inhibitors and epilepsy surgery can reduce the burden of illness and the effectiveness of these therapies should be a focus of further research. Care for individuals with TSC should be organized through specialized TSC centers and their effectiveness at reducing the burden of illness and costs should be investigated. Lastly, finding common tests and protocols to assess the burden of illness in TSC would facilitate research and comparison in this heterogeneous and multifaceted disorder.

## Data Availability

The datasets analysed during the current study available from the corresponding author on reasonable request.
